# Relationship of polymorphisms and haplotype in interleukin-16 and adiponectin gene with late-onset Alzheimer’s disease risk

**DOI:** 10.18632/oncotarget.16297

**Published:** 2017-03-16

**Authors:** Honglei Yin, Yuzhen Zhang, Linlin Hua, Jinfeng Li, Zhilei Zeng, Xiaopeng Yang, Bin Gong, Shuang Geng, Yajun Liu, Hui Zhang, Yanqiu Liu, Jing Zhao, Yunliang Wang

**Affiliations:** ^1^ Department of Neurology, PLA 148 Hospital, Zi Bo, Shandong, China; ^2^ Department of Neurology, Second Affiliated Hospital of Zhengzhou University, Zhengzhou, Henan, China

**Keywords:** interleukin-16, adiponectin, single nucleotide polymorphism, interaction, haplotype

## Abstract

**Aims:**

To investigate the impact of Interleukin-16 (*IL- 16*) and Adiponectin (*ANP*) gene single nucleotide polymorphisms (SNPs), gene- gene interactions and haplotype on late-onset Alzheimer’s disease (LOAD) risk.

**Methods:**

Hardy-Weinberg equilibrium (HWE), haplotype and pairwise linkage disequilibrium (LD) analysis were investigated by using SNPstats (available online at http://bioinfo.iconcologia.net/SNPstats). Generalized multifactor dimensionality reduction (GMDR) was used to examine interaction among 4 SNPs, odds ratio (OR) and 95% confident interval (95%CI) were calculated by logistic regression model.

**Results:**

LOAD risk was significantly higher in carriers of rs266729- G allele than those with CC genotype (CG+ GG *versus* CC), OR (95%CI) =1.61 (1.26-1.96), and higher in carriers of rs1501299- T allele, OR (95%CI) = 1.62 (1.32-2.12), lower in carriers of rs4072111- T allele, adjusted OR (95%CI) =0.65 (0.44-0.93). We also found a significant gene- gene interaction between rs266729 and rs4072111. Participants with CG or GG of rs266729 and CC of rs4072111 genotype have the highest LOAD risk, OR (95%CI) = 2.62 (1.64 -3.58). Haplotype containing the rs266729- G and rs1501299- T alleles were associated with increased LOAD risk, OR (95%CI)= 1.83 (1.32- 2.43), and haplotype containing the rs1131445- C and rs4072111- T alleles were associated with decreased LOAD risk, OR (95%CI)= 0.53 (0.18- 0.95).

**Conclusions:**

We concluded that rs266729 and rs1501299 minor alleles were associated with increased LOAD risk, but rs4072111 minor allele was associated with decreased LOAD risk. We also found that interaction involving rs266729 and rs4072111, and haplotype combinations were associated with LOAD risk.

## INTRODUCTION

Alzheimer’s disease (AD) was a kind of diseases occurred in middle and old age [1], and was associated with some neurological symptoms and cognitive problems, including memory impairment, leading by neurodegeneration or synapse loss leading to and other cognitive problems [2]. Currently, there were a total of 6 million AD patients in China [3]. Clinically, late-onset AD (LOAD) is more common type of AD and the heritability for susceptibility to LOAD was predicted nearly 80% [4]. The etiology and pathogenesis for LOAD were still not clear, and study indicated that LOAD was influenced by interactions between genetic factors and environmental factors [5, 6]. So it is necessary to find and validate biomarkers for AD prevention, especially for LOAD.

Inflammation plays a main role in AD pathogenesis, and the inflammation irritants including damaged tissues and β-amyloid plaque [7]. Interleukin-16 (*IL-16*) is one type of gene, encoded pro- inflammatory cytokines [8]. Studies [9] indicated that *IL-16* levels increased in AD patients, confirming that *IL-16* may play an important role in the progression of AD [9]. The human *IL-16* gene could encode pleiotropic cytokine, which was a modulator of T cell activation [10]. However, to date, just two previous studies [11- 12] were conducted on the association between *IL-16* gene single nucleotide polymorphisms (SNPs) and AD risk, but just one study focused on the correlation of rs4072111, rs1131445 polymorphisms and LOAD risk [11]. There was growing evidence demonstrating the association of adiponectin (*ANP*) gene SNPs with circulating adiponectin levels. rs266729and rs1501299 were two common SNPs. Some studies [13, 14] have reported the association between *ANP* gene polymorphisms and some metabolic diseases, such as insulin resistance and type 2 diabetes, but just one study [15] have focused on the association between *ANP* gene polymorphisms and LOAD.

In consideration of the limited number of study on association between *ANP* and *IL- 16* gene and LOAD, in this study, we aimed to investigate the impact of *ANP* and *IL- 16* gene SNPs, additional gene- gene interaction and haplotype combination on LOAD risk.

## MATERIALS AND METHODS

### Participants

**Figure 1:**
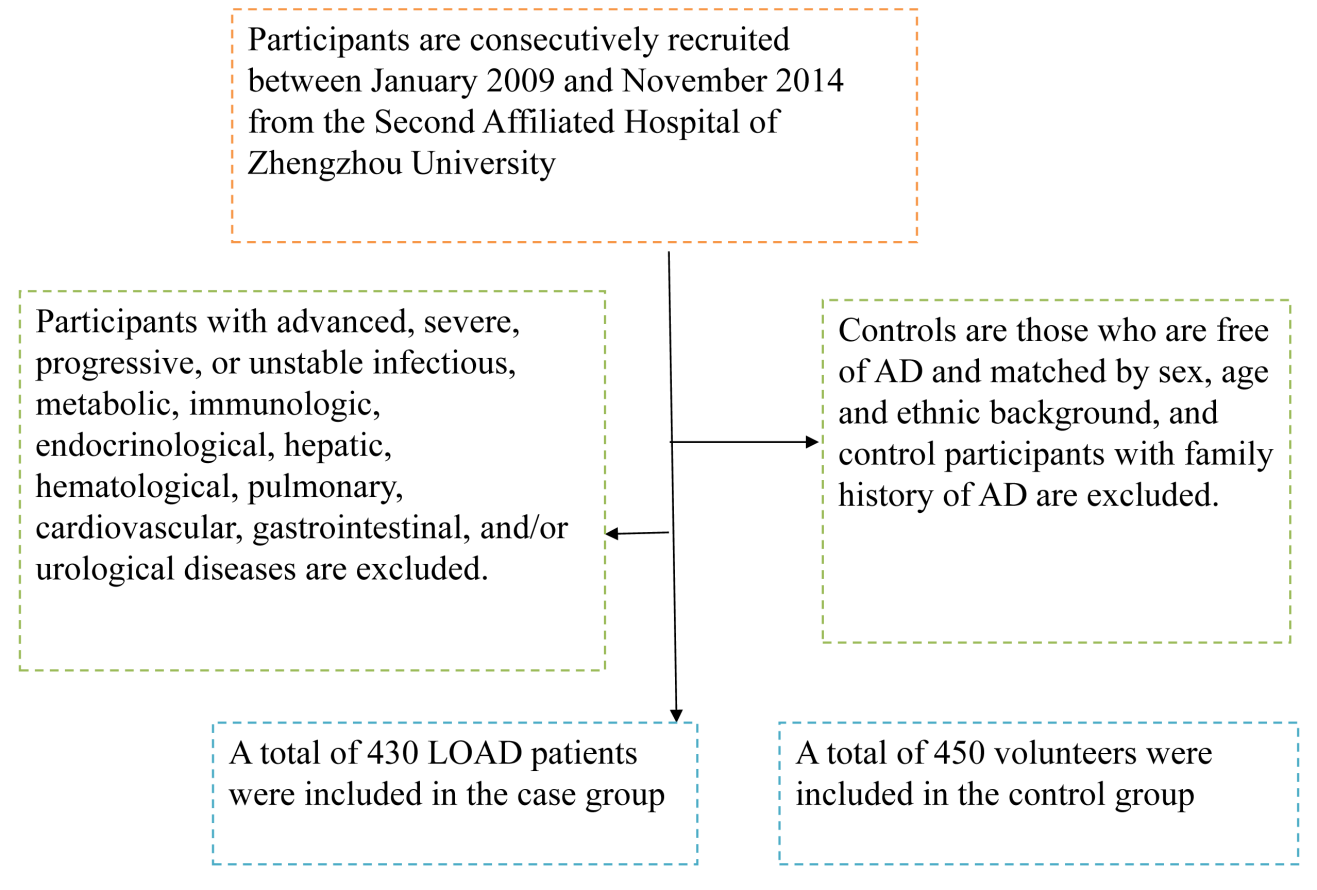
A flowchart on study population selection and exclusion

This is a case-control study. Participants are consecutively recruited between January 2009 and November 2014 from the Second Affiliated Hospital of Zhengzhou University. Clinical diagnosis of probable AD was made according to the revised criteria of National Institute of Neurological and Communicative Disorders and Stroke/ Alzheimer’s Disease and Related Disorders Association (NINCDS/ ADRDA) [16], participants with advanced, severe, progressive, or unstable infectious, metabolic, immunologic, endocrinological, hepatic, hematological, pulmonary, cardiovascular, gastrointestinal, and/or urological diseases are excluded (Figure 1). At last, a total of 430 LOAD patients are included in the study, controls are those who are free of AD and matched by sex, age and ethnic background, and control participants with family history of AD are excluded. The selection and exclusion details could be found in our previous study [17].

### Data collection

Data on demographic information, mini-mental state examination (MMSE), educational year, lifestyle risk factors, smoking and drinking status, prevalence of stroke, prevalence of diabetes and family history of AD for all participants are obtained using a questionnaire administered by trained staffs. Body weight, height and waist circumference (WC) are measured, and body mass index (BMI) are calculated. Blood samples are collected in the morning after at least 8 hours of fasting. All plasma and serum samples are frozen at -80°C until laboratory testing. Plasma glucose is measured using an oxidase enzymatic method. The concentrations of HDL cholesterol and triglycerides are assessed enzymatically using an automatic biochemistry analyzer (Hitachi Inc., Tokyo, Japan) and commercial reagents. Plasma ANP concentration was measured using Adiponectin ELISA kit (Shanghai Huzhen Biological Technology Co., Ltd. China).

### Genomic DNA extraction and genotyping

SNPs within the *ANP* and *IL-16* gene are selected according to the following methods: 1) SNPs, which have been reported associations with AD and were not been well studied; 2) SNPs, the minor allele frequency (MAF) of which were more than 5%. At last, two SNPs of *ANP* gene and two SNPs of *IL- 16* gene are selected for genotyping in the study: rs266729, rs4072111, rs1501299 and rs1131445. Genomic DNA is extracted from EDTA-treated whole blood, using the DNA Blood Mini Kit (Qiagen, Hilden, Germany) according to the manufacturer’s instructions. Genotyping of these SNPs were performed using polymerase chain reaction and restriction fragment length polymorphism (PCR-RFLP) analysis. PCR primer sequences for each polymorphism are shown in Table 1. The PCR reactions were carried out in a final volume of 25 μl containing: 10 × PCR buffer, 4.5 mMMgCl2 (Roche, Germany), 0.4 mM of each dNTP (Fermentas, Germany), 10 pmol of each primer, 30 ng template DNA, 1 U Taq DNA polymerase (Roche, Germany) and sterile distilled water up to 25 μl. Amplification conditions started with an initial denaturation step of 5 min at 94 °C, followed by 35 cycles of 40 s denaturation (94 °C), 30 s annealing (56 °C) and 40 s extension (72 °C), ended by a final extension for 5min (72 °C).

**Table 1:** Description and primer sequences for 4 SNPs used for PCR analysis

SNP ID	Chromosome	Functional Consequence	Major/ minor alleles	Probe sequence
*ANP* gene				
rs266729	3:186841685	Upstream variant 2KB	C/ G	Forward: 5′- ACTTGCCCTGCCTCTGTCTG-3′ Reverse: 5′-CCTGGAGAACTGGAAGCTG-3′
rs1501299	3:186853334	Intron variant	G/ T	Forward: 5′- GGCTCAGGATGCTGTTGCTG-3′ Reverse: 5′-AGGGATGAGGGTGAAGATGGGA-3′
*IL- 16* gene				
rs1131445	15:81309441	Downstream variant 500B, utr variant 3 prime	T/ C	Forward: 5′-GAGATCATTCACTCATACATCTGG-3′ Reverse: 5′-TCATATACACGCTGGTTCCTTCTG-3′
rs4072111	15:81285798	Missense, nc transcript variant	C/ T	Forward: 5′-CACTGTGATCCCGGTCCAGTC-3′ Reverse: 5′-TTCAGGTACAAACCCAGCCAGC-3′

### Statistical analysis

The means and standard deviations were calculated for normally distributed continuous variables, and percentages were calculated for categorical variables. The categorical data were analyzed using χ^2^ test, and continuous variables were analyzed using Student’s t test. Hardy-Weinberg equilibrium (HWE), haplotype analysis and pairwise linkage disequilibrium (LD) analysis were investigated by using SNPStats (available online at http://bioinfo.iconcologia.net/SNPstats). Logistic regression was performed to investigate association between SNP and LOAD by dominant and co- dominant models. All reported *P*-values were two-tailed, and those less than 0.05 were considered statistically significant.

Generalized multifactor dimensionality reduction (GMDR) [18] was used to analysis the interaction among 4 SNPs, cross-validation consistency, the testing balanced accuracy, and the sign test, to assess each selected interaction were calculated. Permutation testing is also conducted to gain empirical P values of prediction accuracy as a benchmark based on 10,000 shuffles. The cross-validation consistency score is a measure of the degree of consistency with which the selected interaction is identified as the best model among all possibilities considered. Testing-balanced accuracy is a measure of the degree to which the interaction accurately predicts case-control status, and yields a score between 0.50 (indicating that the model predicts no better than chance) and 1.00 (indicating perfect prediction). Finally, the sign test, or permutation test (providing empirical *P*-values), for prediction accuracy can be used to measure the significance of an identified model.

## RESULTS

A total of 880 participants (514 males, 366 females) were selected, including 430 LOAD patients and 450 control subjects. The mean age of all participants was 81.7 ± 15.9 years old. The cases have the higher alcohol- drinking rate than controls. The means of FPG and TG were significantly higher in cases and controls, but the mean of HDL was lower in cases and controls. The mean of *ANP* concentrations was higher in controls than that in cases (Table 2).

**Table 2:** General characteristics of 880 study participants in case and control group

Variables	Case group (*n*=430)	Normal group (*n*=450)	*p*-values
Age (year)	81.4±16.1	82.3±15.7	0.401
Males, *N* (%)	246 (57.2)	268(59.6)	0.480
Smoke, *N* (%)	151 (35.1)	145(32.2)	0.364
Alcohol consumption, *N* (%)	188 (43.7)	160 (35.6)	0.013
WC (cm)	89.2±19.8	87.7±19.4	0.257
BMI (kg/m^2^)	25.1±8.9	24.8±9.1	0.621
FPG (mmol/L)	5.8±1.6	5.5±1.9	0.012
TG (mmol/L)	1.4±0.8	1.3± 0.7	0.048
TC (mmol/L)	4.6±0.8	4.5±0.9	0.082
HDL (mmol/L)	1.21±0.65	1.34±0.63	0.002
Stroke	16 (3.72)	20 (4.44)	0.255
MMSE (scores)	15.16±5.51	29.12±4.97	<0.001
Diabetes	36 (8.37)	43(9.56)	0.539
Educational year	7.5±3.12	7.8±3.31	0.167
ANP (mg/L)	3.65±1.06	5.62±1.23	<0.001

Note: Means± standard deviation for age, WC, BMI, FPG, TC, TG, HDL-C and ANP; Abbreviations:TC, total cholesterol; HDL, high density lipoprotein; FPG, fast plasma glucose; TG, triglyceride; WC, waist circumference; BMI, body mass index; MMSE, mini-mental state examination; ANP, Adiponectin. Those *P-* values less than 0.05 were considered statistically significant.

The frequencies for rs266729- G allele and rs1501299- T allele in *ANP* gene were significantly higher in LOAD cases than that in control group (30.4% *vs*19.4%, 32.6% *vs*19.9%), and T allele of rs4072111 in *IL- 16* was significantly lower in LOAD cases than that in control group (19.9 % *vs*29.9%). Logistic regression analysis showed that LOAD risk was significantly higher in carriers with rs266729- G allele than those with CC genotype (CG+ GG *versus* CC), adjusted OR (95%CI) = 1.61 (1.26-1.96), and higher in carriers with rs1501299- T allele than those with GG genotype (GT+ TT *versus* GG), adjusted OR (95%CI) = 1.62 (1.32-2.12). In addition, we also found LOAD risk was also significantly lower in carriers with rs4072111- T allele than those with CC genotype (CT+ TT *versus* CC), adjusted OR (95%CI) = 0.65 (0.44-0.93). (Table 3)

**Table 3:** Genotype and allele frequencies of 4 SNPs between case and control group

Gene/ SNP	Genotypes and Alleles	Frequencies N (%)		OR(95%CI)*	*P*- values	*P*- values for HWE test in controls
		Case (n=430)	Control (n=450)			
*ANP* gene						
rs266729	Codominant					
	CC	213(49.5)	294 (65.3)	1.00		0.550
	CG	173(40.2)	137 (30.5)	1.53(1.22-1.85)	<0.001	
	GG	44(10.3)	19 (4.2)	2.10(1.43-2.94)	<0.001	
	Dominant					
	CC	213(49.5)	294 (65.3)	1.00		
	CG +GG	217(50.5)	156 (34.7)	1.61(1.26-1.96)	<0.001	
	Allele, G (%)	261(30.4)	175(19.4)			
rs1501299	Codominant					
	GG	201(46.7)	289(64.2)	1.00		0.953
	GT	178(41.4)	143(31.8)	1.57(1.25-1.98)	<0.001	
	TT	51(11.9)	18(4.0)	2.05(1.51-2.72)	<0.001	
	Dominant					
	GG	201(46.7)	289(64.2)	1.00		
	GT+TT	229(53.3)	161(35.8)	1.62(1.32-2.12)	<0.001	
	Allele, T (%)	280(32.6)	179(19.9)			
*IL- 16* gene						
rs1131445	Codominant					
	TT	251(58.4)	240(53.3)	1.00		0.898
	TC	156(36.3)	178(39.6)	0.75(0.47-1.09)	0.107	
	CC	23(5.3)	32(7.1)	0.67(0.32-1.03)	0.092	
	Dominant					
	TT	251(58.4)	240(53.3)	1.00		
	TC+CC	179(41.6)	210(46.7)	0.73(0.44-1.08)	0.103	
	Allele, C (%)	202(23.5)	242(26.9)			
rs4072111	Codominant					
	CC	281(65.3)	229(50.9)	1.00		0.079
	CT	127(29.5)	173(38.4)	0.68(0.47-0.93)	0.0012	
	TT	22(5.2)	48(10.7)	0.56(0.26-0.91)	<0.001	
	Dominant					
	CC	281(65.3)	229(50.9)			
	CT+TT	149(34.7)	221(49.1)	0.65(0.44-0.93)	0.001	
	Allele, T (%)	171(19.9)	269(29.9)			

*Adjusted for gender, age, smoking and alcohol status, BMI, WC, FPG, TC, TG, HDL, educational year, prevalence of stroke, prevalence of diabetes. Those *P-* values less than 0.05 were considered statistically significant.

We investigate the impact of the interaction among 4 SNPs within *ANP* and *IL- 16* gene on LOAD risk by using GMDR analysis. We found a significant two-locus model (*p* = 0.0100) involving rs266729 and rs4072111, and the cross-validation consistency of this model was 10/ 10, and the testing accuracy was 60.72%. Participants with CG or GG of rs266729 and CC of rs4072111 genotype have the highest LOAD risk, compared to participants with CC of rs266729 and CT or TT of rs4072111 genotype, OR (95%CI) = 2.62(1.64 -3.58), after covariates adjustment for alcohol consumption status, FPG, TG and HDL (Table 5).

**Table 4:** Best gene–gene interaction models, as identified by GMDR

Locus no.	Best combination	Cross-validation consistency	Testing accuracy	*p*-values *
2	rs266729 rs4072111	10/10	0.6072	0.0100
3	rs266729 rs4072111 rs1501299	9/10	0.5590	0.0547
4	rs266729 rs4072111 rs1501299 rs1131445	8/10	0.5399	0.3770

*Adjusted for gender, age, smoking and alcohol status, BMI, WC, FPG, TC, TG, HDL, educational year, prevalence of stroke, prevalence of diabetes. Those *P-* values less than 0.05 were considered statistically significant.

**Table 5:** Interaction analysis for rs266729 and rs4072111 by using logistic regression

rs266729	rs4072111	OR (95% CI)*	*P*-values
CC	CT or TT	1.00	-
CG or GG	CT or TT	1.18 (1.04 -1.87)	0.026
CC	CC	1.83 (1.48-2.69)	<0.001
CG or GG	CC	2.62 (1.64 -3.58)	<0.001

*Adjusted for gender, age, smoking and alcohol status, BMI, WC, FPG, TC, TG, HDL, educational year, prevalence of stroke, prevalence of diabetes. Those *P-* values less than 0.05 were considered statistically significant.

Pairwise LD analysis between SNPs was measured, and D′ value between rs266729 and rs1501299 was 0.826, D′ value between rs1131445 and rs4072111 was 0.861. Haplotype containing the rs266729- G and rs1501299- T alleles were associated with a statistically increased LOAD risk (OR = 1.83, 95%CI = 1.32- 2.43, *P* < 0.001) (Table 6), and haplotype containing the rs1131445- C and rs4072111- T alleles were associated with a statistically decreased LOAD risk (OR = 0.53, 95%CI = 0.18- 0.95, *P* = 0.012) (Table 7).

**Table 6:** Haplotype analysis on association between *ANP* gene and LOAD risk

Haplotypes	rs266729	rs1501299	Frequencies		OR(95%CI)	*p*-values*
			Case group	Control group		
H1	C	G	0.4601	0.5567	1.00	--
**H2**	**G**	**G**	0.2267	0.2131	1.13 (0.81– 1.62)	0.592
H3	C	T	0.2135	0.1821	1.27 (0.90 - 1.74)	0.602
H4	G	T	0.0997	0.0481	1.83 (1.32 – 2.43)	<0.001

*Adjusted for gender, age, smoking and alcohol status, BMI, WC, FPG, TC, TG, HDL, educational year, prevalence of stroke, prevalence of diabetes.

Those *P-* values less than 0.05 were considered statistically significant.

**Table 7:** Haplotype analysis on association between *IL- 16* and LOAD risk

Haplotypes	rs1131445	rs4072111	Frequencies		OR(95%CI)	*p*-values*
			Case group	Control group		
H1	**T**	C	0.5103	0.4367	1.00	--
H2	**C**	C	0.2624	0.2805	0.67 (0.35 – 1.02)	0.091
H3	**T**	T	0.1926	0.2101	0.72 (0.49 - 1.06)	0.328
H4	**C**	T	0.0347	0.0727	0.53 (0.18 – 0.95)	0.012

*Adjusted for gender, age, smoking and alcohol status, BMI, WC, FPG, TC, TG, HDL, educational year, prevalence of stroke, prevalence of diabetes. Those *P-* values less than 0.05 were considered statistically significant.

## DISCUSSION

In the current study, we found that higher LOAD risks were significantly associated with rs266729- G allele and rs1501299- T allele than those with GG genotype. In addition, we also found that lower LOAD risk was associated with rs4072111- T allele. To date, the relationship between *ANP* gene polymorphism and LOAD risk was not well known, Li et al [15] firstly indicated that the susceptibility to LOAD was higher in carriers of the rs266729- G allele or carriers of the rs1501299- T allele. Previously, studies have involved in *ANP* gene polymorphisms and the others phenotypes. Studies have suggested that higher circulating *ANP* concentration was associated with lower AD risk [19, 20]. Recently, Tong et al [21] found that CC allele was associated with lower serum ANP concentrations, and GG genotype was associated with increased metabolic syndrome and insulin resistance risk [13, 22]. In terms of the correlation of LOAD risk with rs1501299, recent studies [14, 23, 24] also found that serum ANP concentrations were lower in carriers of TT allele.

In recent two studies [11, 12], *IL- 16* gene was associated with LOAD risk. Khoshbakht et al [12] suggested that rs11556218 and rs4778889 polymorphisms within *IL- 16* gene have a protective role in the development of sporadic AD in Iranian population. In the other study conducted by Anvar et al [11] suggested that the rs4072111 variation was associated with increased AD susceptibility in an Iranian population. Rosa et al [9] confirmed that *IL-16* proteins may play an important role in progression of neurodegenerative disorders. Regarding to the relationship between rs4072111, rs1131445 and LOAD, the current study was the second study that concluded a positive association between the *IL16*- rs4072111 polymorphism and LOAD risk. However several studies [25- 27] have reported the association between *IL- 16* gene polymorphism and risk of coronary heart disease (CHD) risk in different population. This relation many be another underlying mechanism for LOAD risk reduction by *IL- 16* polymorphism, because CHD was also associated with AD risk factors, including obesity, metabolic syndrome, and insulin resistance.

In this study, LOAD risk is influenced by both *ANP* and *IL- 16* gene, so it is interesting to investigate the impact of gene- gene interaction between the two genes on LOAD risk. In this study, GMDR model was used for interaction detection, because there were no dimensional constraints in this model. We found a significant gene- gene interaction between rs266729 and rs4072111. To our knowledge this is the first study for investigating impact of interaction between ANP and *IL- 16* gene on LOAD risk in Chinese population. Previously, just two studies [17, 28] focused on the impact of gene- gene interaction on AD risk, which was conducted between *APOE* and *PPAR -γ* gene for Spain population and *CYP2J2* and *PPAR -γ* gene for Chinese Han population. The results of this study suggest that *ANP* genetic variants may modify the influence of *IL- 16* gene on AD risk. The underlying mechanisms for this interaction may due to that both SNP were associated with AD risk factors. We also conducted haplotype analysis in *ANP* gene and *IL- 16* gene respectively. We found that haplotype containing the rs266729- G and rs1501299- T alleles in *ANP* gene were associated with a statistically increased LOAD risk, and haplotype containing the rs1131445- C and rs4072111- T alleles in *IL- 16* gene were associated with a statistically decreased LOAD risk

The current study also has some limitations. Firstly, limited number of SNP in *ANP* and *IL- 16* gene are included in current study, and in the future, more SNPs should be included in analysis. Secondly, gene- environment interaction should be investigated in the future studies, such as lifestyle, diet factors and so on. Thirdly, more detailed analysis should be conducted in other populations, for example, the gender and race difference of this relationship.

In conclusion, the results of current study indicated that LOAD risks are significantly higher in carriers with rs266729- G allele than those with CC genotype, and higher in carriers with rs1501299- T allele than those with GG genotype, and lower in carriers with rs4072111- T allele than those with CC genotype. We also found a significant gene- gene interaction between rs266729 and rs4072111, participants with CG or GG of rs266729 and CC of rs4072111 genotype have the highest LOAD risk, compared to participants with CC of rs266729 and CT or TT of rs4072111 genotype. And haplotype containing the rs266729- G and rs1501299- T alleles in *ANP* gene were associated with a statistically increased LOAD risk, and haplotype containing the rs1131445- C and rs4072111- T alleles in *IL- 16* gene were associated with a statistically decreased LOAD risk

## References

[1] Heneka MT, O’Banion MK (2007). Inammatory processes in Alzheimer’s disease. J Neuroimmunol.

[2] Song X, Mitnitski A, Zhang N, Chen W, Rockwood K, The Alzheimer’s Disease Neuroimaging Initiative (2013). Dynamics of brain structure and cognitive function in the Alzheimer’s disease neuroimaging initiative. Journal of neurology, neurosurgery, and psychiatry.

[3] Yang G, Wang Y, Zeng Y, Gao GF, Liang X, Zhou M, Wan X, Yu S, Jiang Y, Naghavi M, Vos T, Wang H, Lopez AD (2013). Rapid health transition in China, 1990-2010: Findings from the Global Burden of Disease Study 2010. Lancet.

[4] Gatz M, Reynolds CA, Fratiglioni L, Johansson B, Mortimer JA, Berg S, Fiske A, Pedersen NL (2006). Role of genes and environments for explaining Alzheimer disease. Arch Gen Psychiatry.

[5] Araria-Goumidi L, Lambert JC, Cottel D, Amouyel P, Chartier-Harlin MC (2002). No association of the HLA-A2 allele with Alzheimer’s disease. Neurosci Lett.

[6] St George-Hyslop PH, Petit A (2005). Molecular biology and genetics of Alzheimer’s disease. C R Biol.

[7] Xia MQ, Hyman BT (1999). Chemokines/chemokine receptors in the central nervous system and Alzheimer’s disease. J Neurovirol.

[8] Bertram L, Tanzi RE (2004). Alzheimer’s disease: one disorder, too many genes? Hum Mol Genet.

[9] Di Rosa M, Dell’Ombra N, Zambito AM, Malaguarnera M, Nicoletti F, Malaguarnera L (2006). Chitotriosidase and inammatory mediator levels in Alzheimer’s disease and cerebrovascular dementia. Eur J Neurosci.

[10] Greig NH, Mattson MP, Perry T, Chan SL, Giordano T, Sambamurti K, Rogers JT, Ovadia H, Lahiri DK (2004). New therapeutic strategies and drug candidates for neurodegenerative diseases: p53 and TNF-alpha inhibitors, and GLP-1 receptor agonists. Ann N Y Acad Sci.

[11] Anvar NE, Saliminejad K, Ohadi M, Kamali K, Daneshmand P, Khorshid HR (2015). Association between polymorphisms in Interleukin-16 gene and risk of late-onset Alzheimer’s disease. J Neurol Sci.

[12] Khoshbakht T, Soosanabadi M, Neishaboury M, Kamali K, Karimlou M, Bazazzadegan N, Khorram Khorshid HR (2015). An Association Study on IL16 Gene Polymorphisms with the Risk of Sporadic Alzheimer’s Disease. Avicenna J Med Biotechnol.

[13] Lin CH, Ho CY, Liu CS, Lin WY, Li CI, Yang CW, Bau DT, Li TC, Lin CC (2012). Influence of adiponectin gene polymorphisms on adiponectin serum level and insulin resistance index in taiwanese metabolic syndrome patients. Chin J Physiol.

[14] Ramya K, Ayyappa KA, Ghosh S, Mohan V, Radha V (2013). Genetic association of ADIPOQ gene variants with type 2 diabetes, obesity and serum adiponectin levels in south Indian population. Gene.

[15] Li W, Yu Z, Hou D, Zhou L, Deng Y, Tian M, Feng X (2015). Relationship between Adiponectin Gene Polymorphisms and Late-Onset Alzheimer’s Disease. PLoS ONE.

[16] Dubois B, Feldman HH, Jacova C, Dekosky ST, Barberger-Gateau P, Cummings J, Delacourte A, Galasko D, Gauthier S, Jicha G, Meguro K, O’Brien J, Pasquier F (2007). Research criteria for the diagnosis of Alzheimer’s disease: revising the NINCDS-ADRDA criteria. Lancet Neurol.

[17] Geng S, Wang Y, Sun Y, Li J, Yin H, Zeng Z, Yang X, Zhang Y, Wang Y (2016). Gene- gene Interaction between CYP2J2 and PPAR - gene on Late-Onset Alzheimer’s disease in the eastern Chinese Han Population. Behav Brain Res.

[18] Lou XY, Chen GB, Yan L, Ma JZ, Zhu J, Elston RC, Li MD (2007). A generalized combinatorial approach for detecting gene-by gene and gene-by-environment interactions with application to nicotine dependence. Am J Hum Genet.

[19] Teixeira AL, Diniz BS, Campos AC, Miranda AS, Rocha NP, Talib LL, Gattaz WF, Forlenza OV (2013). Decreased levels of circulating adiponectin in mild cognitive impairment and Alzheimer’s disease. Neuromolecular Med.

[20] Pákáski M, Fehér A, Juhász A, Drótos G, Fazekas OC, Kovács J, Janka Z, Kálmán J (2014). Serum adipokine levels modified by donepezil treatment in Alzheimer’s disease. J Alzheimers Dis.

[21] Tong G, Wang N, Leng J, Tong X, Shen Y, Yang J, Ye X, Zhou L, Zhou Y (2013). Common variants in adiponectin gene are associated with coronary artery disease and angiographical severity of coronary atherosclerosis in type 2 diabetes. Cardiovasc Diabetol.

[22] Zadjali F, Al-Yahyaee S, Hassan MO, Albarwani S, Bayoumi RA (2013). Association of adiponectin promoter variants with traits and clusters of metabolic syndrome in Arabs: family-based study. Gene.

[23] Chu H, Wang M, Zhong D, Shi D, Ma L, Tong N, Zhang Z (2013). AdipoQ polymorphisms are associated with type 2 diabetes mellitus: a meta-analysis study. Diabetes Metab Res Rev.

[24] Mtiraoui N, Ezzidi I, Turki A, Chaieb A, Mahjoub T, Almawi WY (2012). Single-nucleotide polymorphisms and haplotypes in the adiponectin gene contribute to the genetic risk for type 2 diabetes in Tunisian Arabs. Diabetes Res Clin Pract.

[25] Huang H, Zeng Z, Zhang L, Liu R, Li X, Qiang O, Zhang Q, Chen Y (2013). The association of interleukin-16 gene polymorphisms with susceptibility of coronary artery disease. Clin Biochem.

[26] Tong Z, Li Q, Zhang J, Wei Y, Miao G, Yang X (2013). Association between interleukin 6 and interleukin 16 gene polymorphisms and coronary heart disease risk in a Chinese population. J Int Med Res.

[27] Wu J, Wang Y, Zhang Y, Li L (2011). Association between interleukin-16 polymorphisms and risk of coronary artery disease. DNA Cell Biol.

[28] Combarros O, Rodríguez-Rodríguez E, Mateo I, Vázquez-Higuera JL, Infante J, Berciano J, Sánchez-Juan P (2011). APOE dependent-association of PPAR - genetic variants with Alzheimer’s disease risk. Neurobiol Aging.

